# Brain-Computer Interface (BCI) combined with Virtual Reality Environment (VRE) for inferior limbs rehabilitation in post-stroke subjects

**DOI:** 10.1186/1753-6561-8-S4-P18

**Published:** 2014-10-01

**Authors:** Berthil Longo, Javier Castillo, Teodiano Bastos

**Affiliations:** 1 UFES Universidade Federal do Espirito Santo, Vitória, Brazil

## 

More than 10 million people in the world live with some kind of motion handicap caused by a Central Nervous System (CNS) dysfunction. Stroke is the major cause of this disability in the adult population. Due to the increase of elderlies in the world's population, such index tends to increase. The proposal of this research is to provide a tool for rehabilitation, useful for subjects that suffer from lower limbs movement handicap, acquired by stroke. This tool carries a 3D Virtual Reality Environment (VRE), which emulates the movement of a heath person, using the immersion of the subject through an avatar. The subject´s brain generates commands to control the avatar while conducting the rehabilitation process. The brain waves are captured by an Electroencephalography (EEG) equipment, that information is sent to a computer for processing, which sends the information to the virtual environment to control the avatar, completing, thereby, the Brain-Computer Interface (BCI) tool. This system asks two different tasks for the subject: move the left or right leg, stimulating brain´s areas responsible for each one of those motor activities, implying thereby, in the rehabilitation process. The VRE provides, for the subject, a feedback of his/her motion intentions. The system works as an attractive environment, which motivates the subject to use it, and, at the same time, is useful to evaluate his/her treatment evolution.
